# Thermocouple Sensor Response in Hot Airstream

**DOI:** 10.3390/s25154634

**Published:** 2025-07-26

**Authors:** Jacek Pieniazek

**Affiliations:** Faculty of Mechanical Engeenering and Aeronautics, Rzeszow University of Technology, al. Powstanców Warszawy 12, 35-959 Rzeszow, Poland; jacek.pieniazek@prz.edu.pl

**Keywords:** thermocouple sensor, FEM, dynamic response, sensor model identification

## Abstract

The response of a temperature sensor in a gas stream depends on several heat transfer phenomena. The temperature of the thermocouple’s hot junction in the hot stream is lower than the measured temperature, which causes a measurement error. Compensation for this error and interpretation of the values indicated by the temperature sensor are possible by using a sensor dynamics model. Changes over time of the hot junction temperature as well as the entire thermocouple temperature in a stream are solved using the finite element method. Fluid flow and heat transfer equations are solved for a particular sensor geometry. This article presents a method for identifying a temperature sensor model using the results of numerical modeling of the response to temperature changes of the fluid stream, in which the input and output signal waveforms are recorded and then used by the estimator of a model coefficient. It is demonstrated that the dynamics of a bare-bead thermocouple sensor are well-described by a first-order transfer function. The proposed method was used to study the influence of stream velocity on the reaction of two sensors differing in the diameter of the wires, and the effect of radiative heat transfer on the model coefficients was examined by enabling and disabling selected models. The results obtained at several calculation points show the influence of the stream outflow velocity and selected geometric parameters on the value of the transfer function coefficients, i.e., transfer function gain and time constant. This study provides quantitative models of changes in sensor dynamics as functions of the coefficients.

## 1. Introduction

Measurements with thermocouple sensors are used in industry and in research, especially when dealing with high temperatures and harsh environments. An example of difficult measurement is the determination of the flame temperature. The output signal of the thermocouple results from the commonly known Seebeck phenomenon. The voltage on the sensor output is a function of the temperature of the measurement junction (hot) and the temperature of the reference junction (cold). Although commonly used thermocouple types are standardized [1], new thermocouple materials are expected to be more accurate and less vulnerable in harsh environments [2]. However, the output signal from the temperature sensor is a function of the temperature of the thermocouple weld of the hot junction. This temperature depends on various phenomena, and can differ from the measured temperature when various factors interfere with the heat exchange between the medium for which the temperature is being measured and the thermocouple hot junction. Although thermocouples have been used in measurements for almost two centuries [1], the issue of accuracy is a continuous subject of research. In addition to wire thermocouples, the design of thin-film thermocouples [3,4] is becoming possible thanks to the progress of materials technology.

The measurement of high temperatures is the most demanding case. Particularly in the case of high temperature gases and combustion processes, many different factors can influence the result. One of the methods to reduce interference is careful design of the sensor case, which forms a flow around the sensor and provides a shield that reduces heat transfer by radiation [2,5]. However, these solutions are only possible when measuring a stream width of considerable width, e.g., exhaust gases in the duct behind the combustion chamber or air inside a compressor. Radiation causing a drop in temperature becomes important in high-temperature measurements, of course; however, the requirement for high accuracy in the lower temperature range makes it necessary to also take into account the heat transfer due to radiation [6].

The response of a thermocouple also depends on the diameters, namely, the wires of the thermocouple and the bead. The larger diameter of the thermocouple wire results in greater resistance to parameter changes and extended service life against chemical reactions that occur at high temperatures. The effect of thermocouple size on the response in exhaust gas was studied in [7], showing a significant effect on heat conduction losses. In another study [8], the authors concluded that the exchange by convection is inversely proportional to the diameter of the thermocouple wire. On the other hand, as the diameter of the wires increases, the effective length (i.e., the length of the part of the wires that accumulates heat, which affects the temperature of the bead) increases as well [9]. Compensation of the thermocouple response of a fine-wire thermocouple by optimizing diameters was the subject of the research presented in [10]. This task requires several numerical models in order to find the best dimensions.

The actual sensor characteristics require model identification. Identification from the measurement experiment should also take into account the noise [11]. While simulations can provide clean data, the quality of applied models is an important factor. Experimental measurements of thermocouple parameters are conducted using various method of temperature change, including chambers, burners, and lasers. For example, in [12] presented the use of a high temperature furnace to obtain the time constant of the thermocouple. Dynamic calibration using lasers is a useful method in the case of thin-film thermocouples [13].

Taking into account the observed phenomena, methods of compensation have been proposed by performing measurements with thermocouples of different diameters, then determining the true temperature by appropriate analysis techniques (e.g., extrapolation from a series of obtained results or estimation for two diameters of thermocouples used simultaneously) or by determining the conditions and current value of parameters (current value of the emission factor) [14,15,16,17]. The emissivity factor, which affects heat transfer by radiation, represents a disturbance in the task of measuring the temperature of the flame when hydrocarbons are burned as a result of soot deposition as well as the burning itself. Tests of the flame temperature during hydrocarbon combustion under fire conditions [18] have led to the conclusion that for some fuels the radiation of the flame only covers external radiation with a flame of more than 1 m. The effect of smoke was investigated in [19] using the simulation method. However, in the case of a transparent gas, the temperature and emissions of the surrounding elements are also important. In both of the above-mentioned tests, the flame had a size of over 10 cm, which causes the temperature around the measuring weld to be uniform.

The classical methods of radiant correction use the Nusselt number for actual conditions, although the reported equations vary [8,16,20]. An additional improvement of the correction method was proposed in [21] taking into account heat transfer through the wires. In addition to analytic reduction of measurement error, a method of increasing convective heat transfer as a result of rapid rotation of the thermocouple junction was proposed in [20]. The results showed the influence of velocity, although indirectly and only qualitatively. The variability of the thermocouple time constant with service life was noted in [22], where the authors further showed how to select the diameters of the weld and of the wires in such a way that the thermal inertia coefficient is invariable by a relatively long time. Despite the influence of fluid velocity on the heat transfer coefficient, most studies have assumed the convection factor to be constant. Some researchers have investigated the recovery factor of the temperature sensor as a parameter of particular geometry [23], which is important for total temperature sensors.

It should be noted that research conducted in the field of thermocouple response combines heat transfer, fluid flow, and material properties. Analytical models include simplified 2D models as well as advanced numerical methods such as the finite element method (FEM).

This study concerns numerical experiments involving the thermometric sensor response in a hot stream flow. The influence of selected air stream parameters and the thermocouple sensor parameter are tested via FEM simulation. The proposed experimental methodology provides the data necessary to determine how the sensor response model depends on operating conditions and sensor geometry. Although the simulation environment models use several variable parameters and the solution is obtained through numerical calculations of 3D models of fluid flow and heat transfer, the final results are in the form of first-order dynamics with two factors, namely, time constant and gain. Repetition of the identification procedure for multiple design points shows that these coefficients are presented as functions of velocity. This new methodology demonstrates the feasibility of developing a simple model with variable parameters depending on the measurement conditions.

## 2. Materials and Methods

Our investigations in this paper concern the determination of the influence of the thermocouple parameters and the parameters of the gas stream on the measurement result; in particular, studies are carried out on the influence of the velocity *v*, gas stream diameter $d_{s}$, and diameter of the thermocouple wires $d_{t}$ on the gain and time constant in the thermocouple model as a first-order dynamical system.

**Figure 1:**
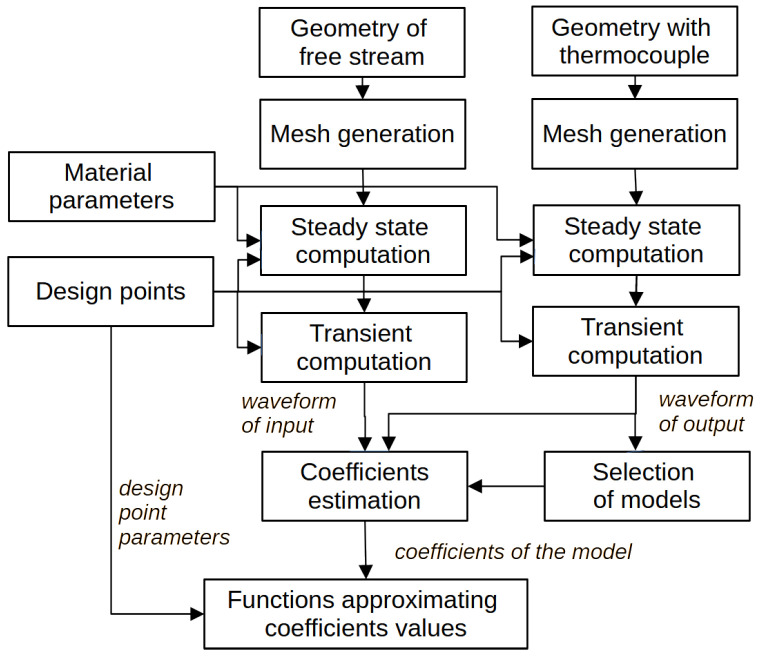
Flow chart of the research methodology.

The method is based on solving equations specific to the phenomena under consideration using the finite element method (FEM). Preparation of geometrical models before performing a numerical experiment is the first step, as shown in Figure 1.

In addition to using the proper set of equations, the accuracy of the reality representation depends on the parameters of the materials. Changes in the parameters, i.e., specific heat and thermal conductivity with temperature, are achieved by introducing appropriate functions in the material properties. In the experiments presented here, a K-type thermocouple is located in flowing air.

To estimate the parameters of the model, the waveforms of the input and output signals are necessary. Two series of experiments provide these data, each containing dozens of design points. After selection of the models, their coefficients of are estimated. The functions that describe the dependence of the coefficients on the velocity are the final result.

### 2.1. Heat Transfer Model of Sensor

Heat transfer between the sensor and its surroundings includes convection, radiation, conduction, and energy accumulation. In Equation (1), the listed components are marked as follows:

- Heat storage process $Q_{1}$ (2), where T(x) is the temperature of the thermocouple along its wires and head *x*, Δm is the mass at *x*, and *c* is the specific heat of the thermocouple wire.
- Heat transfer through convection $Q_{2}$ (3), where ΔA is the outer area, r(x) is the local convection coefficient, and $T_{M}\left(x\right)$ is the temperature of the measured fluid.
- Heat transfer with the environment through radiation $Q_{3}$ (4), where σ is the Stefan–Boltzmann constant, ϵ is the emissivity, and the temperature of the surroundings $T_{o}\left(\phi \right)$ varies depending on the angle ϕ.
- Heat flow through the thermocouple wires $Q_{4}$ (5), where λ is the thermal conduction of the sensor alloys and S(x) is the intersection area.

In addition, the thermal effect of chemical processes occurring on the surface of the thermocouple is denoted by the factor $Q_{5}$; however, this component is neglected in the subsequent experiments.

(1) $$Q_{1}\left(T\right)=Q_{2}\left(T,T_{M}\right)+Q_{3}\left(T,T_{o}\left(\alpha \right)\right)+Q_{4}\left(T\left(x\right)\right)+Q_{5}$$

(2) $$\frac{d\Delta Q_{1}\left(x\right)}{dt}=\Delta m\left(x\right)\cdot c\cdot x\cdot \frac{dT(x)}{dt}$$

(3) $$\frac{d\Delta Q_{2}\left(x\right)}{\left(dt\right)}=\Delta A\left(x\right)\cdot r\left(x\right)\cdot \left(T\left(x\right)-T_{M}\left(x\right)\right)$$

(4) $$\frac{d\Delta Q_{3}\left(x\right)}{dt}=\sigma \cdot \sum_{\phi }\Delta A\left(x\right)\cdot \epsilon \left(\phi \right)\cdot \left(T^{4}\left(x\right)-T_{o}^{4}\left(\phi \right)\right)$$

(5) $$\frac{dQ_{4}\left(x\right)}{dt}=-\lambda \cdot S\left(x\right)\cdot \frac{\partial T(x)}{\partial x}$$

Although Equation (1) shows the simultaneous heat flow through the wires along with the heat transfer between the wires and the environment, the latter process depends on the local conditions; the precise model should consist of 3D partial differential equations with distributed parameters. On the other hand, simplification of the model is possible if one of the factors is dominant, as in the case of temperature measurement of solids and liquids. When high-temperature gases are measured, certain shields and flux-forming cases around the thermocouple reduce other factors and make them negligible compared to convection.

However, despite such a complex model, the relationship between the output signal of the temperature sensor $T_{s}$ and the measured temperature $T_{m}$ comes down to first-order dynamics, which can be stated in the form of Equation (6). In this equation, we have the equivalent mass $m_{eq}$ and mean specific heat $c_{mean}$ of the considered mass along with the mean heat transfer coefficient $r_{mean}$ and equivalent area $A_{eq}$, which are parameters of heat transfer between the sensor and the medium for which the temperature is being measured. In addition, $\Delta T_{m}$ is the temperature error due to heat loss, which can be stated as a coefficient *K*, i.e., the gain of the transfer function. Although Equation (6) is simple, its coefficients are impossible to determine analytically, as they depend not only on the dimensions and properties of the material but also on all of the phenomena described by Equation (1) as well as on the actual flow and environmental conditions.

(6) $$\frac{m_{eq}\cdot c_{mean}}{r_{mean}\cdot A_{eq}}\cdot \frac{dT_{s}}{dt}=T_{m}-\Delta T_{m}-T_{s}=K\cdot T_{m}-T_{s}$$

### 2.2. Identification of the Sensor Model

The response of the temperature sensor is the result of heat transfer processes. The time response of an unshielded thermocouple corresponds to the first-order inertia. The full shield increases the time constant as a result of a significant increase in the heat capacity and also increases the order of the model. Assuming the use of an unshielded thermocouple, the first-order dynamics in the form of a transfer function (7) obtained from Equation (6) are used. The obtained results confirm this choice, as presented below. The transfer function is the quotient of the Laplace transform of the sensor temperature signal $T_{s}$ (output) over the Laplace transform of the measured temperature $T_{m}$ (input), in which τ is the time constant, *K* is the gain, and *s* is independent variable. The time constant is provided by the dependence (8).

(7) $$G\left(s\right)=\frac{T_{s}\left(s\right)}{T_{m}\left(s\right)}=\frac{K}{\tau s+1}$$

(8) $$\tau =\frac{m_{eq}\cdot c_{mean}}{r_{mean}\cdot A_{eq}}$$

If K=1, then the steady-state thermocouple junction reaches the measured temperature perfectly; however, taking into account that the components of Equation (1) include the heat loss due to radiation and conduction, values of *K* less than 1 are expected.

The coefficients in the sensor model (7) may be estimated by the identification process. Identification consists of the following steps:

1. Determination of the parameters of the experiment, taking into account the assumed model and the properties of the tested object.
2. Experiment execution, during which waveforms of the input signal and output signal are recorded.
3. Estimation of model coefficients.
4. Verification to determine the match between the model’s response and that of the tested object.

The basic condition for obtaining correct data for estimation is to obtain a permanent excitation [24]. In the case of a simple model such as (7), a step change is a sufficient excitation.

An experiment for a particular set of tested parameters consists of establishing an initial steady airflow ($T_{0}=293$ K), then applying the step change of the stream temperature. The final temperature of the incoming stream is $T_{1}=1100$ K. The model’s output signal is the average temperature of the thermocouple weld. Due to the specificity of the study, the input signal does not consist of any temperature obtained in the main experiment; instead, additional experiments were carried out under the same conditions as in the main experiment except with the thermocouple removed. The waveform of the average temperature at the location of the thermocouple weld is the input signal for estimation. This method of experimentation removes the influence of the sensor on the fluid flow, and ultimately on the temperature distribution.

The estimation was performed using the n4sid algorithm [25] implemented in MATLAB software R2024b [26]. As this results in the discrete transfer function [24], conversion to the continuous transfer function is applied and the coefficients in the model (7) are extracted.

### 2.3. FEM Simulation Environments

**Figure 2:**
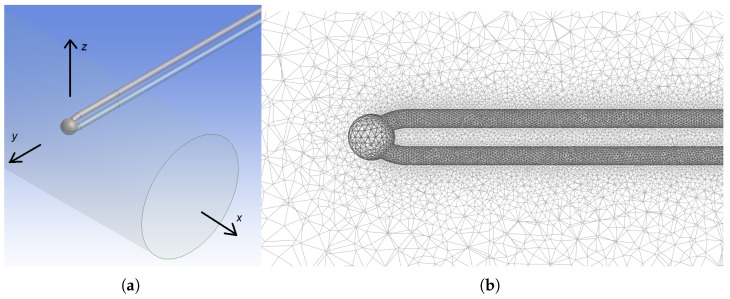
Geometry of the thermocouple sensor: (**a**) inside the domain with coordinate system axes and (**b**) mesh around the thermocouple sensor.

Simulations were conducted in the ANSYS Workbench 2024R2 environment [27,28,29] using FEM. The geometry was developed using DesignModeler. The fluid flow and heat exchange were simulated using Fluent. Several design points were defined for the simulation. The view of the geometry of the thermocouple sensor is presented in Figure 2. The weld head has a diameter 1 mm, which is the same in every design point.

The domain is a cuboid with dimensions of 80 × 80 × 40 mm. The cylinder shown in Figure 2a is inserted into the domain to introduce the stream inlet, which is the circular inlet visible in the projection. This also increases the resolution of the mesh at the interface of the incoming stream with the surrounding air. The sensor is centrally located at a distance of 10 mm from the inlet. The boundary conditions, i.e., the velocity inlet, pressure outlet, and wall, are defined on the corresponding walls of the cuboid.

The mesh details vary among the different zones. A dense mesh grid is necessary inside the thermocouple sensor as well as near the solid–fluid interface in order to increase the spatial resolution of the computation in the space relevant to the study. The mesh view in the area near the sensor is presented in Figure 2b. The weld is located in the cross-section of the sphere between the wires.

The simulation of heat transfer and heat accumulation uses various material parameters which change with the temperature. The chromel and alumel materials which are part of thermocouple type K are defined in the Fluent 2024R2 setup, as are the air parameters. The setup exported from Fluent is included in the Supplementary Materials.

### 2.4. Simulation Methodology

**Figure 3:**
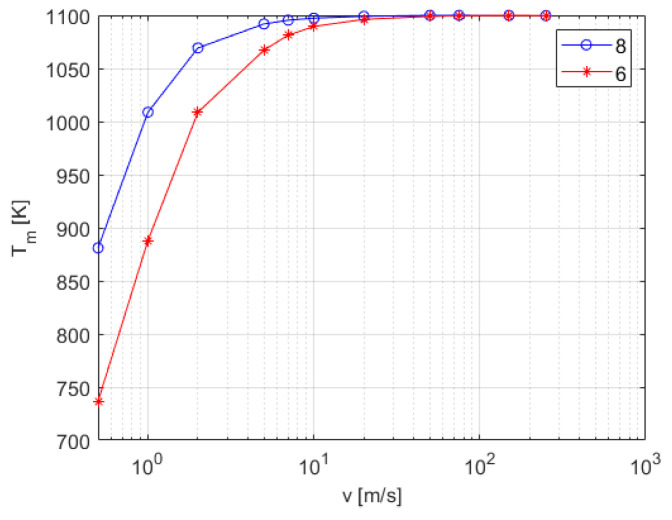
Final temperature of the airstream at the position of the thermocouple as a function of velocity for two initial stream diameters (diameters [mm] in legend).

**Figure 4:**
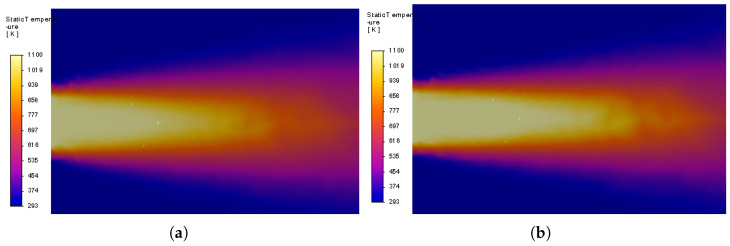
Temperature distribution on the xy plane without the thermocouple: (**a**) v = 5 m/s and (**b**) v = 75 m/s.

The experiments without a sensor in the fluid flow deliver reference waveforms in the form of the real temperature of the stream $T_{m}\left(t\right)$ at the location of the sensor under the conditions defined by the stream diameter and its initial velocity. The final value of the temperature at this location $T_{2}=T_{m}\left(t_{final}\right)$ was obtained by transient simulation as a function of the air stream velocity, with the results presented in Figure 3. Two samples of the temperature distribution at the xz intersection at $t_{final}$ are presented in Figure 4.

**Figure 5:**
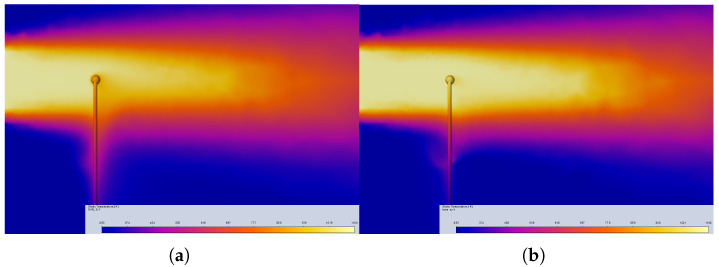
Temperature distribution on the xy plane around the thermocouple: (**a**) v = 5 m/s and (**b**) v = 75 m/s.

Two samples of the temperature distribution with the thermocouple are presented in Figure 5. Comparison of the temperature distributions presented in Figure 4 and Figure 5 obtained for the same stream diameter and same velocities illustrate the distortion of the stream caused by thermocouple insertion. The airflow around the sensor bead depends on the design point parameters, and is influenced by the thermocouple wires. The wires (at the bottom) also cause heat transfer towards their locations.

**Figure 6:**
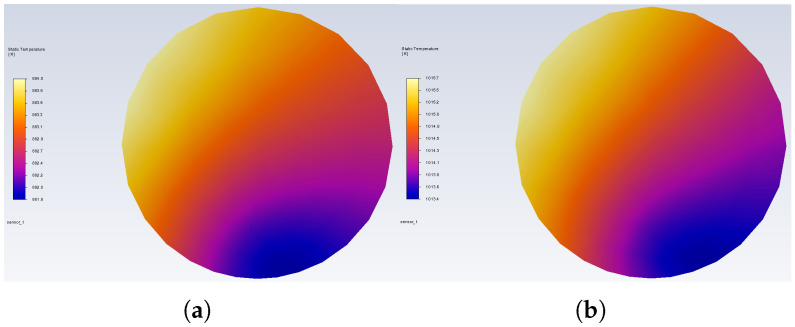
Temperature distribution on the xy plane inside the thermocouple weld: (**a**) v = 5 m/s and (**b**) v = 75 m/s.

The temperature distribution that occurs in the weld obtained under the same conditions as for Figure 5 is presented in Figure 6. The observed temperature differences are in the range of 2.5 K for all design points, and the highest and lowest temperature locations are similar. Such a small deviation is expected as a result of airflow disturbances caused by the thermocouple wires.

## 3. Results

Taking into account the temperature distribution in the weld, the primary result of the main experiment is the waveform of the average temperature of the thermocouple weld. The waveform of this temperature is taken over the simulation time. These results are used to identify coefficients in the model (7) and finally to determine the dependence of *K* and τ from the measurement conditions.

### 3.1. Sensor Responses

**Figure 7:**
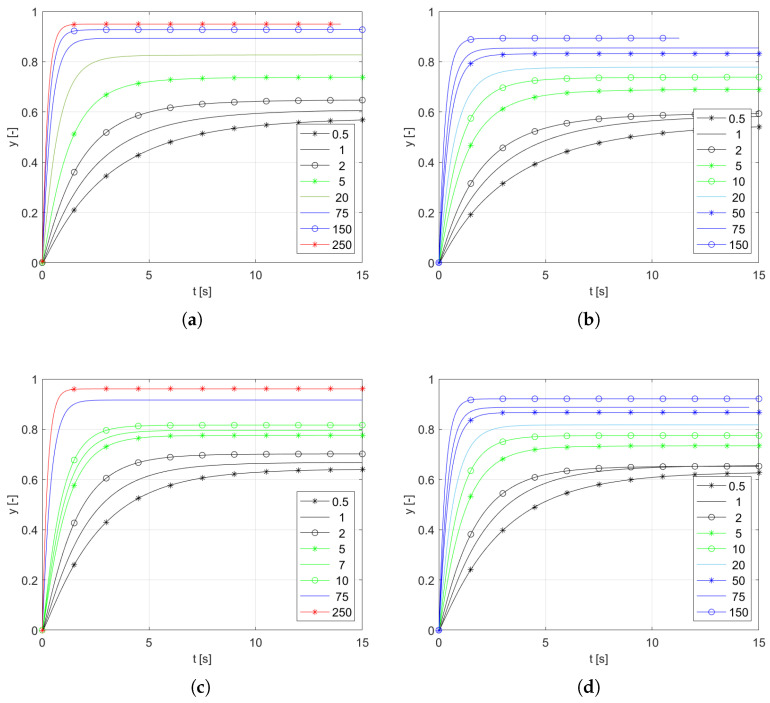
Sample responses: (**a**) inlet diameter 8 mm, thermocouple wire 0.4 mm; (**b**) inlet diameter 6 mm, thermocouple wire 0.4 mm; (**c**) inlet diameter 8 mm, thermocouple wire 0.3 mm; (**d**) inlet diameter 6 mm, thermocouple wire 0.3 mm.

The output signals for the two diameters of the incoming stream (6 mm and 8 mm) and for the two diameters of the thermocouple wires (0.4 mm and 0.3 mm) are recorded in the range of velocity from 0.5 m/s to 250 m/s. The initial temperature is $T_{0}=293$ K, while the final input temperature is $T_{1}=1100$ K. Due to the dispersion of the flux, the temperature around the thermocouple is affected by the flow conditions. Instead of a temperature value, the value of *y* calculated from the dependency (9) is shown in Figure 7. Using the variable *y* instead of the temperature *T* shows the output change due to the step excitation, and is normalized to the amplitude of the actual excitation obtained during the experiment.

(9) $$y\left(t\right)=\frac{T\left(t\right)-T_{0}}{T_{2}-T_{0}}$$

The decreasing time constant τ and increasing coefficient *K* with increasing velocity are easily observable effects.

### 3.2. Identification Results

The results of experiments both with and without the thermocouple provided the data for subsequent estimation of the model parameters. The parameters of the transfer function are presented in Table 1 (thermocouple wire 0.3 mm) and Table 2 (thermocouple wire 0.4 mm). The fit is a normalized measure of how the model response matches the estimation data [26]. Values close to one hundred percent mean that the deviation from the first-order dynamics is small.

**Figure 8:**
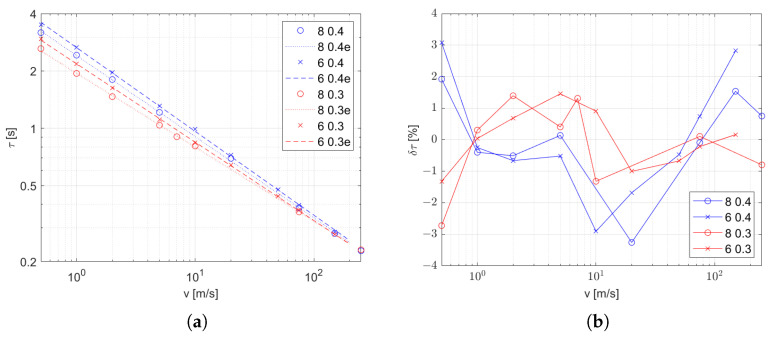
Transfer function coefficients τ with various *d* and $d_{wire}$, as described in the legend: (**a**) as function of inlet velocity (marked points) and approximation (dashed line) and (**b**) relative error of approximation.

When analyzing the results, it was found that the impact of the velocity is strongly nonlinear, which is consistent with the influence of the velocity on the heat exchange coefficient. The heat exchange coefficient is the most variable parameter in (10), and is the main influence on the time constant; however, the obtained points in the logarithmic chart presented in Figure 8a are arranged on the line. Taking this observation into account, the approximation function candidate has the form in (10).

(10) $$\tau \left(v\right)=a\cdot v^{b}$$

The coefficients in the function presented in Table 3 are computed as a minimal-square approximation. The values of the relative approximation error are shown in Figure 8b.

**Figure 9:**
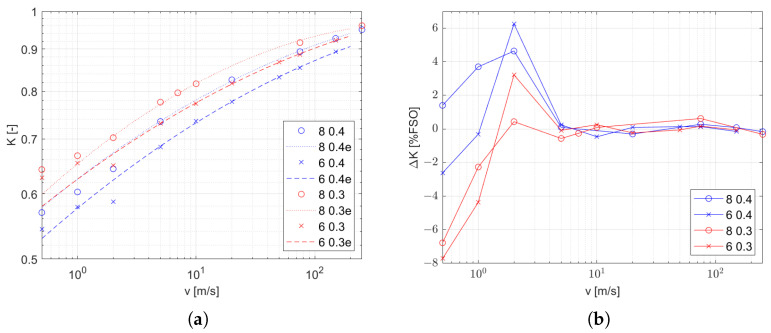
Transfer function gain *K* with various *d* and $d_{wire}$, as described in the legend: (**a**) as function of inlet velocity (marked points) and approximation (dashed line) and (**b**) relative error of approximation.

The gain *K* is nonlinear, but is also shown in the logarithmic chart in Figure 9a. However, note that in the velocity range from 5 m/s, the characteristic is regular for all experimental conditions. The second-degree polynomial, which results in the following form of the function (11), is chosen for approximation within this range of velocities.

(11) $$K\left(v\right)=a\cdot \exp(a_{1}\cdot \ln\left(v\right)+a_{2}\cdot {\left(\ln\left(v\right)\right)}^{2})$$

The approximating function parameters for two diameters of the thermocouple wires and two diameters of the hot inlet are presented in Table 4. The values of the approximation error as a percentage of the full-scale output over the full velocity range are shown in Figure 9b.

### 3.3. Effect of Radiation Heat Transfer

**Figure 10:**
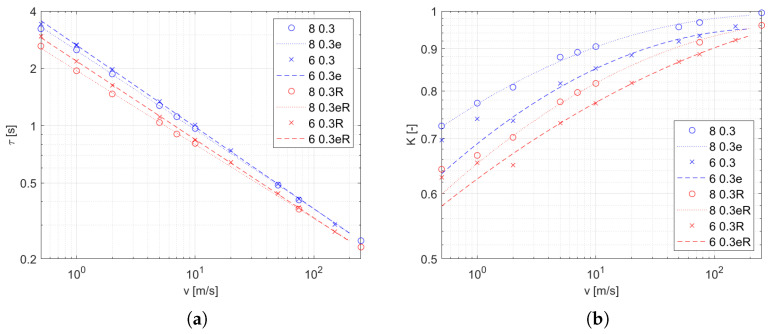
Transfer function coefficients as function of inlet velocity (marked points) and approximation (dashed line). Experimental conditions: *d* in the legend and $d_{wire}=0.3$ mm. The results with active radiation equations are marked in red, while those without the radiation model are in blue. (**a**) τ (**b**) *K*.

**Figure 11:**
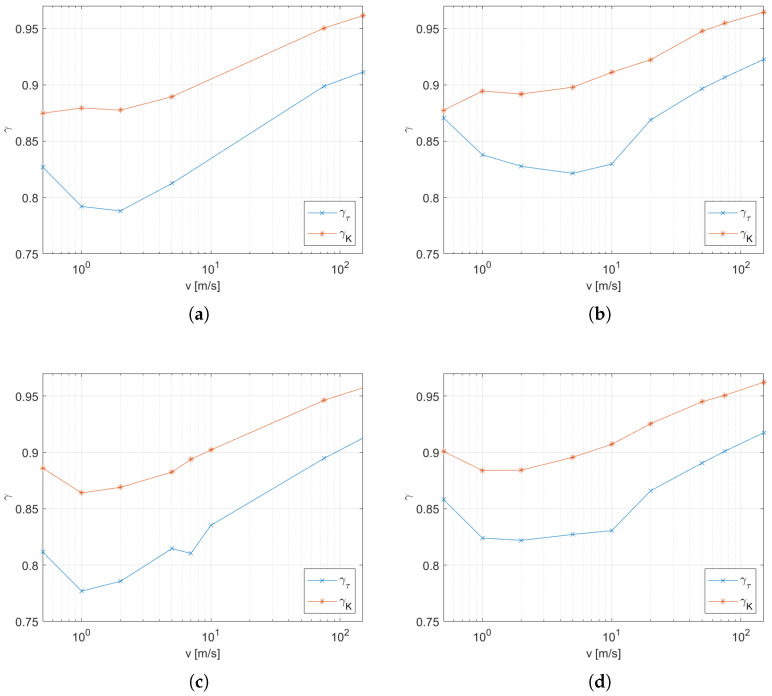
Changes of the parameters τ and *K* caused by radiation heat transfer as described by the γ parameter defined in (12): (**a**) inlet diameter 8 mm, thermocouple wire 0.4 mm; (**b**) inlet diameter 6, mm thermocouple wire 0.4 mm; (**c**) inlet diameter 8 mm, thermocouple wire 0.3 mm; (**d**) inlet diameter 6 mm, thermocouple wire 0.3 mm.

In addition to the main experiment, a simulation was carried out with the radiation model turned off. The resulting coefficients were obtained in the same way as in the main experiment, and the results are shown in Figure 10 as blue dots and lines. In addition, the previously obtained coefficients for a thermocouple with the same diameter of wires and with the same inlet diameter are presented as red points and lines. These graphs make it easy to compare the results with the complete model to those without the radiation model. The parameters for the functions (10) and (11) are listed in Table 3 and Table 4. A significant decrease in the gain *K* along with a decrease in τ are observable effects due to radiative heat transfer. To quantify this change, the variables γ (12) are defined as the quotient of the values of a given factor obtained with and without the thermal radiation model, indicated by the suffixes *R* and nR, respectively. The coefficients of the time constant $\gamma_{\tau }$ and gain $\gamma_{K}$ for the four conditions as a function of the airstream velocity are shown in Figure 11.

(12) $$\begin{array}{c} \gamma_{\tau }=\frac{\tau_{R}}{\tau_{nR}}\\ \gamma_{K}=\frac{K_{R}}{K_{nR}} \end{array}$$

## 4. Discussion

As a result of our studies of a bare thermoelectric temperature sensor in the flow of a hot gas, it turns out that the behavior of such a sensor is well described by the first-order inertial model, which is confirmed by the values of fitting coefficient near 100% (Table 1 and Table 2). The coefficients of this model, that is, the time constant τ and the gain *K*, are strongly dependent on the conditions of heat transfer with the medium for which the temperature is being measured. The speed at which the sensor responds depends on τ. Based on the obtained results, we were able to obtain a model for the influence of the flow velocity on this coefficient which has the same form regardless of the flow conditions. However, the coefficients of this function are influenced by both the size of the stream and the thickness of the thermocouple wires, and the influence of these factors decreases with increase in the flow rate (Figure 8 and Figure 10a). The fitting deviation of the function τ(v) is in the range of ±3%. An incorrect value of the time constant causes a temperature error, the maximum value of which is specified by the dependency (see (A5) in Appendix A). The dynamic error of the measurement due to this model mismatch is in the range of ±1.1% when −3% and +3% are considered as limits of the error of the estimated τ.

The second coefficient is the gain, which directly affects the value of the measurement error. Based on the characteristics we obtained (Figure 9 and Figure 10b), it can be stated that the size of the stream and the thickness of the thermocouple wires affect the gain to a greater extent than for τ and that this effect is irregular. The quadratic function on a logarithmic scale of variables that results in the form (11) provides a good approximation of the effect of the velocity on gain in the velocity range above 5 m/s. The resulting gain deviation is within ±0.7 percent of the full output.

In the low-velocity range (below 5 m/s), the effect of flow velocity on the value of *K* varies for each of the cases we considered. This cannot be associated with a change in the nature of convection, as the value of the Richardson number is below 0.03. The observed effects mean that the development of the characteristics of *K*, which is crucial for the reconstruction of the measured value, requires a greater scope of research than for τ, especially for low velocities and small stream sizes.

The presented research methodology for studying the characteristics of the temperature sensor in the gas flow uses advanced computational methods to model physical phenomena. Thanks to the ability to turn on and off selected models, it is possible to determine the impact of a selected phenomenon on the behavior of the sensor. In this study, we determined the effect of radiative heat transfer on sensor temperature in this way. The results shown in Figure 10 indicate a reduction of both τ and *K* due to radiative heat transfer. The first effect is caused by an increase in the intensity of heat transfer between the thermocouple and the hot stream. On the other hand, the loss of heat at high temperature is due to exchange with the low temperature environment (set as $T_{0}$ on all walls of domain except for the hot airstream inlet) causing a decrease in the steady-state temperature, i.e., it leads to a decrease in *K*. This effect becomes more intense with decreasing velocity of the airstream as the intensity of convection decreases; however, the dispersion of the stream causes a decrease in temperature at the thermocouple’s location (Figure 3), which also reduces the intensity of the radiant heat losses. These two effects explain the shape of $\gamma_{\tau }$ and $\gamma_{K}$ in Figure 11, where the gradient dγ/dv decreases for the low-velocity range and also changes its sign at the boundary. It is worth noting that the sensitivity to flow velocity is very small at low velocities and for small streams (Figure 11d).

However, it should be noted that our experiments assumed the emissivity of the thermocouple surface ϵ=1. Under real conditions, the possible values depend on the surface condition obtained in production as well as on the influence of environmental factors during the period of use. Therefore, the results presented in Figure 11 can be treated as the lower end of the range of possible values of the change factor of the model parameter with an emissivity between 0 and 1.

The presented results were obtained for the assumed parameters of the mesh. The mesh density influences computation time, accuracy, and stability of a solver, and typically represents a compromise between accuracy and time of computation. To test the effect of the mesh, an experimental comparison of responses with two velocities (1 m/s and 150 m/s) was performed to see how the mesh refinement affected the results. The increase of the mesh density was achieved by changing the growth rate parameter from 1.2 to 1.05. As a result, the number of elements increased eight times, resulting in an approximately eightfold increase in the calculation time. The resulting deviation of of *K* is 0.4 % (*v* = 1 m/s) and 0.5 % (*v* = 150 m/s), while the deviation of τ is about 0.1 % (*v* = 1 m/s) and 0.9 % (*v* = 150 m/s). The increase in differences with velocity is expected to be due to an increase in the Courant number; nonetheless, the implicit solver method appears to be robust for the case under consideration.

## 5. Conclusions

This study illustrates how the results of computational fluid dynamics and heat transfer are used to obtain a sensor model. By incorporating these methods into the identification process, it is possible to determine the properties of the sensor according to the conditions under which the measurement is performed. In particular, the presented results show the influence of velocity and heat transfer by radiation. The obtained relationships indicate a relatively regular change of the time constant over the entire range of the velocities studied. On the other hand, the sensitivity, i.e., gain in the transfer function, shows irregularities in the low-velocity range. These irregularities depend on the dimensions of the sensor as well as the size of the hot airstream.

Having obtained a model of the transducer under certain conditions, it is possible to reconstruct the actual measured value while also taking into account the dynamics of the response. The model and the dependence of its parameters on the air velocity make it possible to compensate for the response from the actual measurement. The obtained results show that the behavior of a bare thermoelectric sensor, although generally following the first-order dynamics, depends on many factors; hence, the problem arises of comparing results obtained in different studies and based on use in different conditions. Because the results presented here refer to a specific geometry of thermoelectric sensor, there is also the question of how changes in geometry (bead-type or butt weld; straight or curved wires) affect the parameters of the model. This requires further research, in which the proposed methodology will certainly be useful.

While the presented methodology is applicable in the area of determining sensor models, which can be used to improve accuracy through numerical compensation, it should be noted that the obtained results used several transient simulations. Each simulation of the design point takes several hours (depending of course on the available computing power), which is due to the size of the model grid and the number of equations needed to describe the physical phenomena.

The results themselves can provide information about how physical phenomena affect measurements carried out with temperature sensors. Unlike a real experiment, simulations allow for selective study of the influence of various physical factors and phenomena. Results obtained under different conditions also provide clues about how to modify the measurement conditions in order to reduce the influence of selected factors.

## Figures and Tables

**Table 1:** Experimental parameters and estimated coefficients of the transfer function (thermocouple wire diameter = 0.3 mm).

*d* [mm]	*v* [m/s]	*K*	τ [s]	*fit* [%]
6	0.5	0.63	2.94	99.7
6	1.0	0.65	2.18	99.5
6	2.0	0.65	1.63	99.4
6	5.0	0.73	1.11	98.9
6	10.0	0.77	0.84	98.7
6	20.0	0.82	0.64	98.6
6	50.0	0.87	0.44	98.2
6	75.0	0.89	0.37	98.3
6	150.0	0.92	0.28	98.7
8	0.5	0.64	2.62	99.2
8	1.0	0.67	1.94	98.8
8	2.0	0.70	1.47	98.4
8	2.0	0.70	1.47	98.4
8	5.0	0.78	1.04	97.9
8	7.0	0.80	0.90	97.5
8	10.0	0.82	0.81	97.7
8	75.0	0.92	0.36	97.5
8	250.0	0.96	0.23	98.2

**Table 2:** Experimental parameters and estimated coefficients of transfer function (thermocouple wire diameter = 0.4 mm).

*d* [mm]	*v* [m/s]	*K*	τ [s]	*fit* [%]
6	0.5	0.54	3.49	99.6
6	1.0	0.58	2.66	99.4
6	2.0	0.59	1.97	99.3
6	5.0	0.68	1.31	99.2
6	10.0	0.74	0.99	99.1
6	20.0	0.78	0.72	99.1
6	50.0	0.83	0.48	98.9
6	75.0	0.85	0.39	98.8
6	150.0	0.89	0.29	98.6
8	0.5	0.57	3.18	99.7
8	1.0	0.60	2.42	99.6
8	2.0	0.64	1.80	99.5
8	5.0	0.73	1.21	98.9
8	20.0	0.83	0.70	98.5
8	75.0	0.89	0.38	98.0
8	150.0	0.93	0.28	98.0
8	250.0	0.95	0.23	97.6

**Table 3:** Parameters of function (10) approximating the data in Table 1 and Table 2 with and without the heat radiation model.

$d_{\mathrm{wire}}$ [mm]	*d* [mm]	*a*	*b*
With radiation heat transfer			
0.4	8	2.411	−0.426
0.4	6	2.653	−0.440
0.3	8	1.948	−0.388
0.3	6	2.182	−0.411
Without radiation heat transfer			
0.4	8	3.003	−0.451
0.4	6	3.177	−0.455
0.3	8	2.482	−0.416
0.3	6	2.641	−0.429

**Table 4:** Parameters of the function (11) approximating the data in Table 1 and Table 2 with and without the heat radiation model.

$d_{\mathrm{wire}}$ [mm]	*d* [mm]	*a*	$a_{1}$	$a_{2}$
With radiation heat transfer				
0.4	8	0.626	0.111	−0.00642
0.4	6	0.576	0.118	−0.00616
0.3	8	0.653	0.118	−0.00881
0.3	6	0.625	0.106	−0.00572
Without radiation heat transfer				
0.4	8	0.748	0.0672	−0.00325
0.4	6	0.633	0.122	−0.00928
0.3	8	0.771	0.0867	−0.00752
0.3	6	0.69	0.115	−0.0102

## Data Availability

All data are contained within the article or Supplementary Materials.

## Supplementary Materials

The following supporting information can be downloaded at: https://www.mdpi.com/article/10.3390/s25154634/s1.

## Appendix A. Maximal Dynamic Error Caused by Estimation Error

An error caused by the dynamic system (7) due to erroneous $\tau_{e}=\tau \cdot \left(1+\beta \right)$ is the result of differences between the responses of the sensor model and the real sensor. The general form of response to the input T(t) is provided by (A1), where ∗ is the convolution. The worst case, i.e., reaching the maximum dynamical error, is caused by the step response difference (A2), where $T_{a}$ is the amplitude of the step input.

(A1) $$T_{m}\left(t\right)=\frac{K}{\tau }\cdot \left(e^{-t/\tau }\right)*T\left(t\right)$$

(A2) $$\Delta T_{m}\left(t\right)=T_{a}\cdot K\cdot \left(e^{t/\tau }-e^{t/\tau_{e}}\right)$$

The maximum value of the error is at the time, when $\frac{d\Delta T_{m}\left(t\right)}{dt}=0$. After differentiating the function (A2) and further rearrangement, we obtain $t_{max}$ (A3).

(A3) $$t_{max}=\tau \cdot \frac{ln(1+\beta )}{\beta }$$

Defining the maximum relative error of the measurement (A4), its value is calculated by substituting $t_{max}$ into the function (A2), assuming K=1.

(A4) $$\delta T_{max}=\frac{\Delta T_{m}\left(t_{max}\right)}{T_{a}}$$

A relative error β of τ results in the maximum relative dynamic error according to the dependency in (A5).

(A5) $$\delta T_{max}=-\beta \cdot {\left(1+\beta \right)}^{-1-1/\beta }$$
