# Supplementary material for: Thermocouple Sensor Response in Hot Airstream
Source: Sensors (Basel). 2025 Jul 26;25(15):4634. doi: 10.3390/s25154634 (PMC12349096; doi:10.3390/s25154634)
Supplement: Supplementary file 1 [file sensors-25-04634-s001.zip › description.pdf]

Raw data for the “Thermocouple response in hot airstream”
Jacek Pieniążek

Raw data being the result of the heat transfer and flow modelling in ANSYS Fluent are recorded into several files numbered in the file name (***xx*** and ***yy***) according to the design point numbers. The parameters of the design points are in the csv files imported from Workbench. The data contains records of input temperature (files 1, 2) and sensor responses (files 3, 4, 5) used as described in the paper.

The temperature response records with and without thermocouple has different sample times and input temperature is recorded for the shorter time because it faster responds. Before using it it is necessary to resample data to the same time step and extend input records by adding an appropriate number of points with value of steady state (last value of the temperature record).

1. DPref.csv

File contains design point data of simulation without thermocouple (ANSYS Workbench formatting)

Columns (comma separated):

1 – “DP” and (***xx*)** number of design point

2, 4 (**vf**) – velocity of the central airstream (both should be the same)

3 (**Rf**) – radius of the central airstream (only for Rf=3 and Rf=4 computed)

5 – final temperature of the step input (1100 K is used)

6 – mean temperature in the area of the sensor (at the end of initial simulation)

7 – temperature at the centre of the sensor (at the end of initial simulation)

8 – mean temperature in the area of the sensor (at the end of main simulation)

9 – temperature at the centre of the sensor (at the end of main simulation)

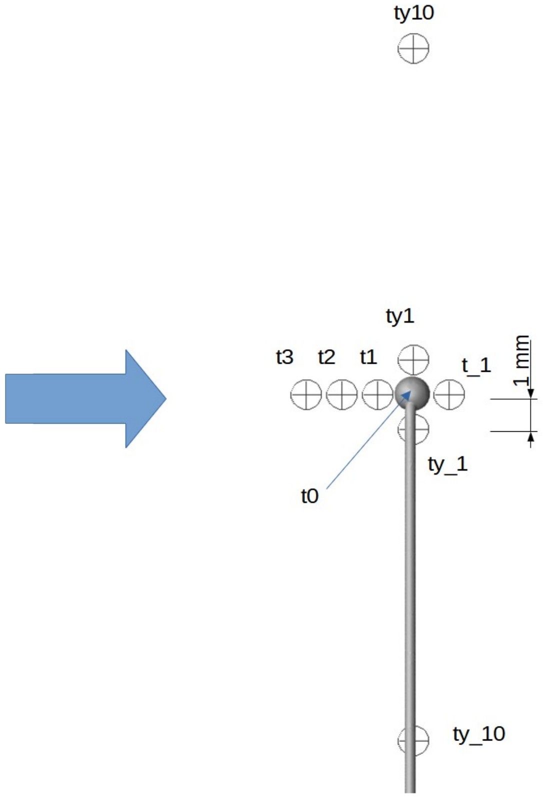

Figure 1. Points where temperature is recorded

2. report-file-rt_(*xx*).out
File contains records from transient modelling of the hot stream without thermocouple
Columns:
"TimeStep" – number of time step
"delta-time" – time step size (0.01 s)
"flow-time" – actual model time
"iters-per-timestep" – no of iterations at time step
temperature at various points (see Figure 1):
"t_1"
"t_sk" – mean temperature in the area of the sensor
"t0" – temperature at the centre point of the sensor location
"t1"
"t2"
"t3"
"ty_1"
"ty_10"
"ty1"
"ty10"

----

3. DP.csv
File contains design point data of main experiment (ANSYS Workbench formatting).
Columns (comma separated):
1 – "DP" and (***yy***) number of design point
2, 3 (**r**) – radius of thermocouple wires (the same for both)
4 – position (not used, always 0)
5 (**Rf**) – radius of the central airstream (only for Rf=3 and Rf=4 computed)
6, 7, 9 – velocity of the central airstream (all should be the same)
8, 10 – final temperature of the step input (both should be the same)

4. report-file-t_11R(*yy*).out
File contains records from transient modelling of the hot stream with thermocouple and radiative heat transfer model **on**
Columns:
"TimeStep" – number of time step
"delta-time" – time step size (0.005 s)
"flow-time" – actual model time
"iters-per-timestep" – number of iterations at time step
temperature at various points (see Figure 1):
"t_1"
"t_sensor" – mean temperature in the area of the sensor
"t0" – temperature at the centre point of the sensor
"t1"
"t2"
"t3"
"ty_1"
"ty_10"
"ty1"
"ty10"

5. report-file-tc_11bR(*yy*).out

File contains records from transient modelling of the hot stream with thermocouple and radiative heat transfer model **off**

Columns:

"TimeStep" – number of time step

"delta-time" – time step size (0.005 s)

"flow-time" – actual model time

"iters-per-timestep" – number of iterations at time step

temperature at various points (see Figure 1):

"t_1"

"t_sensor" – mean temperature in the area of the sensor

"t0" – temperature at the centre point of the sensor

"t1"

"t2"

"t3"

"ty_1"

"ty_10"

"ty1"

"ty10"
